# Case Report: Uremic encephalopathy in acute kidney injury caused by abdominal sepsis

**DOI:** 10.3389/fradi.2026.1875949

**Published:** 2026-07-10

**Authors:** Sergey Karasev, Rustam Talybov, Tatyana Trofimova, Irina Karaseva

**Affiliations:** 1 Department of Neurosurgery the Federal Center of Neurosurgery, Tyumen, Russia; 2 Radiology Department, Regional Clinical Hospital № 2, Tyumen, Russia; 3 Oncology Department, Tyumen State Medical University of the Ministry of Healthcare of Russia, Tyumen, Russia; 4 Radiology Department, First Pavlov State Medical University of St. Petersburg, St. Petersburg, Russia

**Keywords:** abdominal sepsis, acute kidney injury, neuroimaging, uremia, uremic encephalopathy

## Abstract

Uremic encephalopathy encompasses a wide range of central nervous system disorders resulting from acute or chronic kidney damage. The pathogenesis of this syndrome is multifactorial and includes the accumulation of uremic toxins, hormonal imbalance, electrolyte and acid-base imbalance, blood-brain barrier dysfunction, and systemic inflammation. There are no specific clinical or laboratory markers for uremic encephalopathy, and the diagnosis is often made retrospectively, based on the dynamics of neurological status. The diagnostic process is further complicated by the presence of comorbid and overlapping diseases. Consequently, renal replacement therapy is often initiated at an suboptimal time. Neuroimaging plays a key role in confirming the diagnosis, providing high sensitivity in assessing the nature and extent of brain tissue damage. The “lenticular fork sign” is recognized as the most significant MRI marker and can serve as an early and reliable indicator of uremic encephalopathy. The implementation of an interdisciplinary strategy, including a comprehensive laboratory assessment and timely neuroimaging, allows not only to confirm the pathogenetic mechanisms underlying central nervous system damage, but also to promptly adjust therapeutic treatment. This publication presents a clinical case of uremic encephalopathy resulting from acute kidney injury associated with abdominal sepsis in a 50-year-old patient.

## Introduction

Sepsis is defined as a life-threatening condition arising from dysregulation of the host immune response to an infectious agent, which leads to generalized tissue and organ damage with subsequent multiple organ dysfunction (1). According to the World Health Organization, over 48 million cases of sepsis were registered in 2021, with the number of sepsis-associated deaths reaching 11 million, accounting for 19.7% of global mortality (2). Multiple organ dysfunction, occurring in approximately 51% of cases, is the leading complication and primary determinant of adverse outcome in this patient population. The clinical spectrum of organ failure encompasses cardiovascular insufficiency, acute lung injury, acute kidney injury (AKI), hepatic failure, and central nervous system dysfunction (3–7). AKI is recorded in more than 50% of patients with critical sepsis and is associated with a significant 62% increase in the risk of death (8). Rapid progression of renal dysfunction leads to the development of uremic syndrome – a systemic pathological process characterized by the accumulation of endogenous uremic toxins, decompensation of hormonal regulation, severe disturbances of fluid-electrolyte and acid-base homeostasis, altered vascular reactivity, dysfunction of the blood-brain barrier (BBB), and persistent systemic inflammation (9). Collectively, these mechanisms predetermine multiple organ damage, including neurological disorders and severe cerebral complications, which significantly aggravate the course of the septic process. Uremic encephalopathy (UE) is a toxic-metabolic injury to the central nervous system (CNS) that predictably develops in patients with acute or chronic kidney injury (10). This condition is based on a multicomponent pathogenetic mechanism involving metabolic and hemodynamic disturbances, accumulation of uremic toxins in the systemic circulation, BBB dysfunction, disrupted transmembrane water transport, hormonal imbalance, and systemic inflammation accompanied by vascular wall calcification and increased permeability (10). The pathological impact on the brain is exerted via two main pathways: indirectly – through vasculopathy and hemostatic disorders, and directly – through neurotoxicity, induction of neuroinflammation, oxidative stress in glial cells, and BBB disintegration (11). Verification of UE in clinical practice is often retrospective, based on assessment of neurological dynamics after initiation of renal replacement therapy (10, 11). To date, there are no validated clinical-laboratory criteria enabling reliable identification of this condition at early stages in routine practice. An additional factor significantly limiting timely diagnosis is the high comorbidity in this patient group, necessitating complex differential diagnosis with other cerebral disorders and frequently leading to delayed recognition of uremic encephalopathy as the primary pathogenetic driver of neurological deficits (12).

## Case report

A 50-year-old male patient was brought by the emergency medical team to the admission department of the Regional Clinical Hospital No. 2. Upon admission, his condition was assessed as severe, as evidenced by forced positioning and intense pain syndrome. Complaints were characterized by unrelenting, increasing pain in the right iliac region, significantly exacerbated by attempts to change body position. A medical history from relatives and outpatient records, it was established that the patient had not previously been followed by a nephrologist and had no history of chronic kidney disease. Arterial hypertension, diabetes mellitus, chronic heart failure, epilepsy, cerebrovascular diseases, hepatitis, and HIV infection were denied. Allergological history was unremarkable. Smoking (20 pack-years) and occasional alcohol consumption were noted. The regular use of any medications on a permanent basis was denied.

Objectively: clear consciousness, Glasgow Coma Scale (GCS) – 15, pupils D = S, photomotor response brisk, no oculomotor disturbances, facial symmetry preserved, swallowing intact, muscle tone physiological, deep tendon reflexes brisk and symmetrical D = S, no pathological plantar responses, meningeal signs (neck stiffness, Kernig's and Brudzinski's signs) negative. Coordination tests performed satisfactorily given the pain syndrome. No focal neurological deficits were detected. Body temperature up to 38 °C, arterial blood pressure – 100/50 mmHg, respiratory rate – 22 per minute. In the admission department, the on-call surgical team reasonably suspected acute abdomen based on the clinical picture. To verify the diagnosis, a set of clinical-diagnostic measures was carried out, including complete blood count, blood chemistry, urinalysis, abdominal ultrasound, and non-contrast abdominal computed tomography (CT). Based on the combined analysis of imaging data and laboratory findings, a preliminary diagnosis of destructive appendicitis with generalized peritonitis was made. The quick Sequential Organ Failure Assessment (qSOFA) score was 1, indicating low risk of adverse outcome. Emergency surgery was performed, consisting of diagnostic laparotomy, appendectomy, sanitation, and drainage of the abdominal cavity. Intraoperatively, the diagnosis of acute destructive appendicitis and generalized serous-fibrinous peritonitis was confirmed. In the early postoperative period, the patient was observed in the intensive care unit. After extubation and a 24-hour dynamic observation, he was transferred to the surgical department for ongoing inpatient management. On the third postoperative day, deterioration was noted, manifesting as febrile fever up to 38.0–39.5 °C, increasing general weakness, dyspnea, and oliguria. Neurological status at this stage corresponded to mild stupor (GCS 13). Blood chemistry revealed signs of AKI: blood urea nitrogen 4.5 mmol/L, creatinine 185 µmol/L, metabolic acidosis (pH 7.2), and a critical decline in estimated glomerular filtration rate (eGFR) to 25 mL/min/1.73 m^2^. Platelet count was 90 × 10⁹/L, total bilirubin up to 26 µmol/L. Based on clinical-laboratory data, manifestations of AKI with uremic symptoms were established. Infusion therapy with 25% albumin solution and balanced crystalloid solutions was initiated at 30 mL/kg over the first 2 h. The SOFA score was 8. Due to progressive multiple organ failure against the background of abdominal sepsis, the patient was transferred to the intensive care unit.

On the following day, a sharp increase in neurological symptoms was recorded: the patient's condition worsened to coma (GCS 6) refractory to ongoing standard intensive therapy; episodes of generalized seizures occurred. A rapid rise in nitrogenous waste levels was registered: creatinine reached 480 µmol/L, eGFR was 12 mL/min/1.73 m^2^, and anuria persisted for more than 12 h. To verify the etiology of the progressing neurological deficit, a neuroimaging study was performed on the evening of day 4. Brain MRI was performed a Signa Voyager (General Electric) 1.5 T scanner with a standardized protocol, including T1-weighted, T2-weighted, T2-FLAIR, diffusion-weighted images (DWI) with apparent diffusion coefficient (ADC) maps, and SWAN (T2*-gradient echo) sequences in three mutually perpendicular planes with slice thickness of 1–5 mm, without contrast administration. MRI findings (Figure 1) revealed a pathognomonic sign for severe metabolic (particularly uremic) encephalopathies – bilateral symmetric foci of abnormal signal in the basal ganglia, characterized by hyperintensity on T2-WI and T2-FLAIR, with a hyperintense rim of the lentiform nucleus, consistent with the “lentiform fork sign” (LFS). Additionally, combined involvement of subcortical regions of the frontal, parietal, and insular lobes, as well as the thalamus, was detected, manifesting as a combination of vasogenic and cytotoxic edema, indicating a multifocal pattern of cerebral injury with involvement of both cortical and deep nuclear structures. Based on a comprehensive assessment of laboratory findings, severity of neurological status, and the MRI findings, the picture was interpreted as uremic encephalopathy resulting from sepsis-induced AKI. Therapy was adjusted in accordance with the established diagnosis. Further fluid management was guided by the diameter of the inferior vena cava and central venous pressure; a restrictive strategy was adopted, with the main volume loading delivered enterally. This approach allowed target volemic status parameters to be achieved. Renal replacement therapy (RRT) was initiated as continuous veno-venous hemodiafiltration (CVVHDF) with an effluent dose of 35–40 mL/kg/h for 28 h, as a pathogenetically justified method for eliminating uremic toxins and correcting homeostasis.

**Figure 1:**
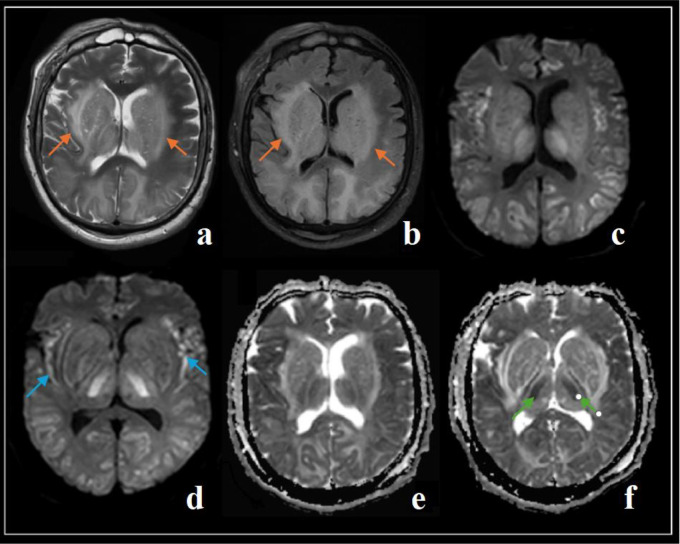
Brain MRI of a patient with uremic encephalopathy: **(a)** T2-WI; **(b)** T2-FLAIR; **(c,d)** DWI; **(e,f)** ADC. Extensive areas of abnormal signal in the caudate and lentiform nuclei bilaterally, demonstrates hyperintense signal on T2/FLAIR images outlining the basal ganglia and producing the “lentiform fork sign” (orange arrows). Additionally, a combination of vasogenic edema (subcortical regions of the frontal, parietal, and insular lobes) (blue arrows) and cytotoxic edema (thalamic regions) (green arrows) is registered.

By the 7th day of treatment, clear positive dynamics were noted. Neurological status improved to stupor (GCS 9) without seizure episodes. A stable tendency toward normalization of renal nitrogen-excretory function against a background of recovering intrinsic diuresis was recorded: creatinine decreased to 150 µmol/L, eGFR increased to 49 mL/min/1.73 m^2^. To objectify the regression of cerebral abnormalities, a follow-up brain MRI was performed using the identical protocol (Figure 2). Comparative neuroimaging analysis revealed marked positive dynamics in the form of significant reduction of zones of cytotoxic and vasogenic edema in the subcortical regions of the frontal, parietal, and insular lobes, thalami, and basal ganglia, which correlated with clinical improvement in neurological status. By the 14th day from the initiation of comprehensive intensive therapy, complete recovery of consciousness was observed (GCS 15, SOFA 2); after resolution of critical AKI and UE manifestations, the patient was transferred to the specialized department for further treatment of the underlying disease and completion of rehabilitation measures. The patient was discharged from the hospital in a compensated state on the 25th day. Upon discharge, the patient reported that the most distressing episodes for him were the complete disorientation and loss of consciousness during the comatose period, and he expressed high appreciation for the efforts of the medical staff directed toward his recovery. During the recovery phase, he did not experience any pain syndrome; however, he noted pronounced asthenia and generalized weakness throughout the first week following emergence from the comatose state. The patient expressed satisfaction with the treatment outcomes and the absence of any neurological deficit at the time of discharge, which enabled him to return to his habitual daily activities without the need for external assistance or additional care.

**Figure 2:**
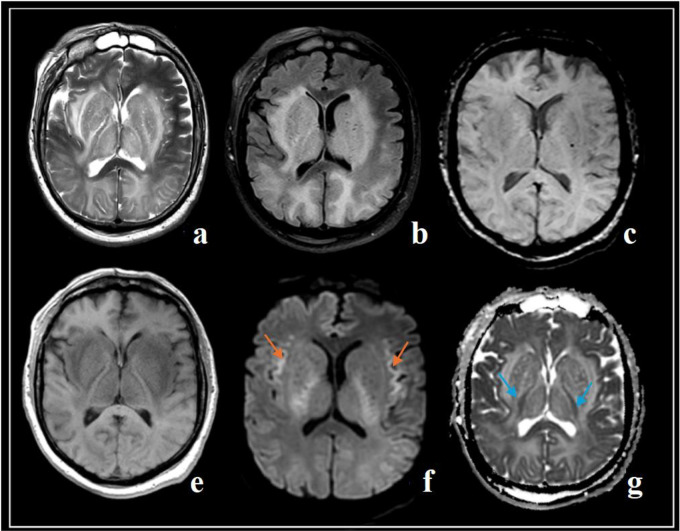
A follow-up brain MRI 7 days after correction of therapy: **(a)** T2-WI; **(b)** T2-FLAIR; **(c)** SWAN; **(d)** T1; **(e)** DWI; **(f)** ADC. Marked regression of cytotoxic (blue arrows) and vasogenic edema (orange arrows) is noted in subcortical regions of the frontal, parietal, and insular lobes, as well as in the basal ganglia and thalami.

A detailed timeline of the case is presented in Table 1.

**Table 1:** Timeline of clinical events, laboratory parameters, and neuroimaging findings.

Time point	Event	GCS	Creatinine, µmol/L	eGFR, mL/min/1.73 m2	Urea, mmol/L	Potassium, mmol/L	pH	Platelets, 10⁹/L	SOFA	Notes
Day 0, 10:00	Admission, emergency surgery	15	85	>90	6.0	4.2	7.38	220	–	qSOFA=1
Day 3	Deterioration, oliguria	12	185	25	18.5	5.1	7.20	90	8	Transfer to ICU
Day 4, morning	Coma, anuria, seizures	6	480	12	35.0	5.8	7.15	85	–	Consultation meeting
Day 4, evening	First MRI (Figure 1) and Initiation of CVVHDF	6	480	12	35.0	5.8	7.15	85	–	Lentiform fork sign detected
Day 7	Follow-up MRI (Figure 2)	9	150	49	12.0	4.5	7.35	150	–	Regression of edema
Day 14	Transfer to specialized hospital ward	15	100	>60	7.0	4.0	7.40	210	2	Consciousness fully recovered
Day 25	Discharge	15	80	>60	5.5	3.9	7.40	250	0	Compensated state

## Discussion

Despite significant advances in intensive care, sepsis remains a global healthcare challenge, characterized by high mortality and a high incidence of multiple organ dysfunction. Its pathogenesis involves a complex cascade of immune reactions, including a hyperinflammatory response, coagulopathy with disseminated intravascular coagulation, and pronounced hemodynamic instability (3, 4). Collectively, these disturbances lead to reduced perfusion of target organs, including the kidneys, thereby contributing to the development of AKI. The occurrence of uremia, caused by ischemic injury and tubular epithelial necrosis, in turn exerts systemic toxic effects, the key element of which is CNS injury with the development of UE (13) (Figure 3). The pathogenetic basis of UE is the complex impact of neurotoxic compounds. Uremic toxins (UTs), such as indoxyl sulfate and p-cresyl sulfate, are potent inducers of oxidative stress, contributing to vascular dysfunction, disruption of blood-brain barrier integrity, neuroinflammation, and neuronal death via pyroptosis, necroptosis, and ferroptosis (14). An additional contribution comes from neurotransmitter imbalance: accumulation of guanidine compounds stimulates N-methyl-D-aspartate (NMDA) receptors, exerting excitotoxic effects, while concurrent inhibition of GABA receptors disrupts the balance between inhibitory and excitatory amino acids (11, 14). The presented clinical case clearly demonstrates how the combination of abdominal sepsis and AKI leads to the rapid manifestation of neurological symptoms, confirming the concept of synergistic damaging effects of systemic inflammation and uremia on brain tissue.

**Figure 3:**
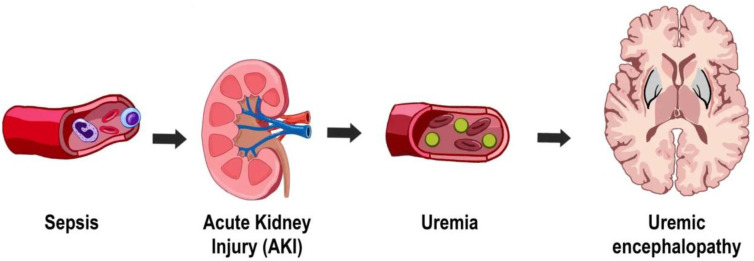
Pathogenetic mechanisms of uremic encephalopathy in sepsis.

Notably, despite an initial qSOFA score of 1, which corresponds to low risk of adverse outcome, the patient developed severe multiorgan dysfunction. This observation highlights a well-known limitation of the qSOFA scale – its low sensitivity in the early stages of sepsis, particularly in patients without comorbidity, in whom compensatory mechanisms may temporarily mask the severity of the condition (15). This case is consistent with the literature indicating that qSOFA should not be used as the sole risk stratification tool, and that clinical suspicion for sepsis-associated AKI and encephalopathy should remain high even with “low” scores.

Neuroimaging, predominantly MRI, which is highly sensitive in assessing the nature and extent of brain involvement, acquires special importance in the diagnosis of UE (16). According to literature analysis, the imaging pattern of UE is represented by three main types: 1) involvement of the basal ganglia; 2) cortical or subcortical involvement; 3) white matter involvement (11). The most frequent finding is cytotoxic and/or vasogenic edema in the basal ganglia, internal capsules, and periventricular white matter. In our observation, the key MRI indicator was the “lentiform fork sign” (LFS), consistent with studies positioning this sign as an early and reliable marker of UE (11, 17, 18). The high vulnerability of the basal ganglia is explained by their high metabolic activity, sensitivity to mitochondrial dysfunction, and the peculiarities of endothelial insufficiency of cerebral vessels against the background of uremia and metabolic acidosis (18, 19). Differential diagnosis of UE poses significant difficulties due to overlap of symptoms with other neurological and metabolic conditions. Thus, during the differential diagnostic workup, posterior reversible encephalopathy syndrome (PRES) was excluded based on the following criteria: the absence of documented sustained arterial hypertension both in the patient's medical history and during the acute phase of the disease, as well as the lack of characteristic lesion localization involving the posterior circulation. An infectious etiology of the encephalopathy was reasonably ruled out by the results of lumbar puncture performed prior to the initiation of antibiotic therapy, in conjunction with negative bacterioscopic findings and negative polymerase chain reaction assays for neurotropic viral agents. The diagnosis of hepatic encephalopathy was not supported, given that transaminase levels (ALT, AST) remained within reference ranges and there were no clinical signs of hepatocellular insufficiency; meanwhile, moderate hyperbilirubinemia was interpreted as a manifestation of systemic inflammatory response and multiorgan dysfunction rather than as a marker of primary hepatic involvement. Metabolic disorders were excluded by means of biochemical screening, which revealed no critical deviations in glycemia or electrolyte balance. Toxic encephalopathies were ruled out on the basis of negative toxicological screening results and the absence of any history of nephrotoxic or psychoactive pharmacological agent intake. Key differential diagnostic criteria UE include a combination of clinical features (presence of renal failure, azotemia, metabolic acidosis) and imaging characteristics (bilateral basal ganglia involvement with LFS and edema pattern) (11). In the presented clinical case, systemic analysis enabled verification of UE, underscoring the combined importance of clinical, laboratory, and radiological data for an accurate diagnosis.

Despite its high diagnostic value, it must be acknowledged that LFS is not absolutely pathognomonic for UE. Similar MRI changes in the basal ganglia region may be observed in conditions such as metabolic acidosis, hypoxic-ischemic injury, Wilson's disease, autoimmune encephalitides, metabolic encephalopathies, as well as in certain drug-induced encephalopathies (11, 20). In this regard, the interpretation of MRI findings should always be performed strictly within the context of the clinical presentation and laboratory data. In our observation, it was precisely the combination of acute renal failure with severe azotemia and metabolic acidosis at the onset of neurological disturbances that constituted the decisive factor in favor of UE, rather than an isolated assessment of the neuroimaging. This underscores the necessity of a comprehensive approach and cautions against interpreting LFS as the sole marker of the disease. An important clinical feature of UE, confirmed in our observation, is its potential reversibility provided that uremia is adequately corrected. Intensive hemodialysis led not only to normalization of creatinine level and a greater than 60% reduction in blood urea nitrogen, but also to marked regression of neurological symptoms and positive dynamics on follow-up MRI. This fact confirms the key role of neutralizing neurotoxic compounds in the treatment of UE and justifies the need for early initiation of renal replacement therapy in patients with sepsis complicated by AKI. Despite the high diagnostic value of MRI, performing this examination in critically ill patients is associated with practical difficulties, including severity of condition, the need for vital function monitoring, and significant scanning time. In this regard, optimizing MRI protocols to reduce examination time and developing clear criteria for selecting septic patients for neuroimaging remain relevant tasks. Implementation of these approaches will allow timely verification of UE, minimize diagnostic errors, and consequently reduce mortality and the incidence of neurological complications in this patient category.

We acknowledge the limitations of the present case report due to the absence of long-term follow-up data. Although complete regression of neurological symptoms and restoration of renal function were achieved during the hospital stay, the assessment of long-term cognitive outcomes and the risk of chronic kidney disease remains beyond the scope of this work and necessitates further longitudinal observation of the patient.

## Conclusion

 The presented clinical case convincingly substantiates the need for a fundamental reconsideration of approaches to managing patients with sepsis. The key predictor of a favorable outcome is not so much the control of the primary infectious focus, but the early diagnosis of sepsis-associated complications – in particular, acute kidney injury and toxic encephalopathy. The use of a multidisciplinary strategy that includes a comprehensive history, thorough analysis of laboratory data, and timely MRI neuroimaging enables not only the verification of the pathogenetic mechanisms of CNS injury but also the timely adjustment of therapeutic tactics. This case confirms that a personalized approach, based on a high index of clinical suspicion among physicians of various specialties, is the determining factor for success in treating this complex patient population.

## Data Availability

The original contributions presented in the study are included in the article/Supplementary Material, further inquiries can be directed to the corresponding author.
